# Supplementary material for: Ndfip1 restricts mTORC1 signalling and glycolysis in regulatory T cells to prevent autoinflammatory disease
Source: Nat Commun. 2017 Jun 5;8:15677. doi: 10.1038/ncomms15677 (PMC5465375; doi:10.1038/ncomms15677)
Supplement: Supplementary Information — Supplementary Figures [file ncomms15677-s1.pdf]

Supplementary Figure 1

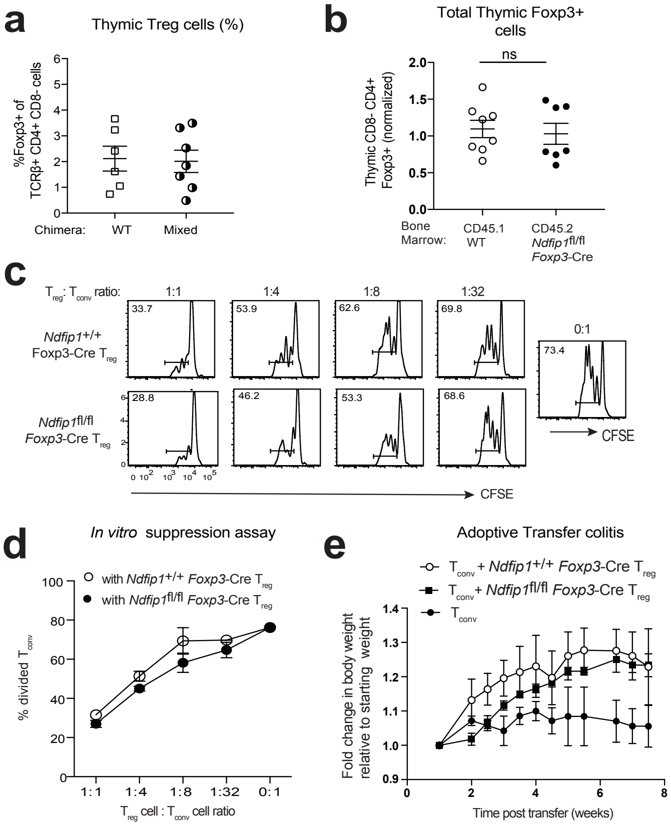

Supplementary Figure 1. *Ndfip1*-deficient T_{reg} cells have normal thymic numbers and peripheral suppression.

(**a-b**) *Ndfip1*$^{+/+}$*Foxp3*-Cre (WT) only or WT/ *Ndfip1*$^{fl/fl}$ *Foxp3*-Cre mixed bone marrow chimeras were analyzed 6-8 weeks after reconstitution. (**a**) Percentages of thymic T_{reg} cells for WT only or WT/ *Ndfip1*$^{fl/fl}$ *Foxp3*-Cre mixed chimeras. Gated on singlets, live cells, FSCa by SSCa lymphocytes. (**b**) Normalized congenic contributions to total T_{reg} cells in the thymi of mixed chimera animals. Normalization to account for reconstitution bias was done by dividing the percentage of CD45.1^{+} WT cells or CD45.2^{+} *Ndfip1*-deficient cells within the pool of total T_{reg} cells (TCRβ^{+}CD4^{+}CD8^{-} Foxp3^{+}) by the percentages within the pool of total CD4+ single positive cells (TCRβ^{+}CD4^{+}CD8^{-} Foxp3^{-}). (**c-e**) *Ndfip1*-deficient regulatory T cells have normal *in vitro* and *in vivo* suppression of wild type T_{conv} cells. (**c-d**) *In vitro* suppression of congenically marked (CD45.1), CFSE-labeled, WT CD4^{+} T_{conv} cells by congenic WT T_{reg} cells or *Ndfip1*$^{fl/fl}$ *Foxp3*-Cre T_{reg} cells (**c**) CFSE dilution of T_{conv} cells after 4 days in culture (**d**) Quantification of the percentage of T_{conv} cells that diluted CFSE in culture (**e**) Weight changes during an *in vivo* suppression assay using CD45.1 CD4^{+} T_{conv} cells and CD45.2 WT or *Ndfip1*$^{fl/fl}$ *Foxp3*-Cre T_{reg} cells. Error bars indicate mean ± SEM. Each dot represents an individual mouse. Male donors were used for mixed bone marrow chimeras (a-b). These chimeras are also shown in Figure 4. (c-e) represents data from at least 3 male animals of each genotype. (e) Represents one experiment. P values were determined by a paired *t* test (**a-b**) and student's *t* test (**c-e**) * $p<0.05$ ** $p<0.01$

Supplementary Figure 2

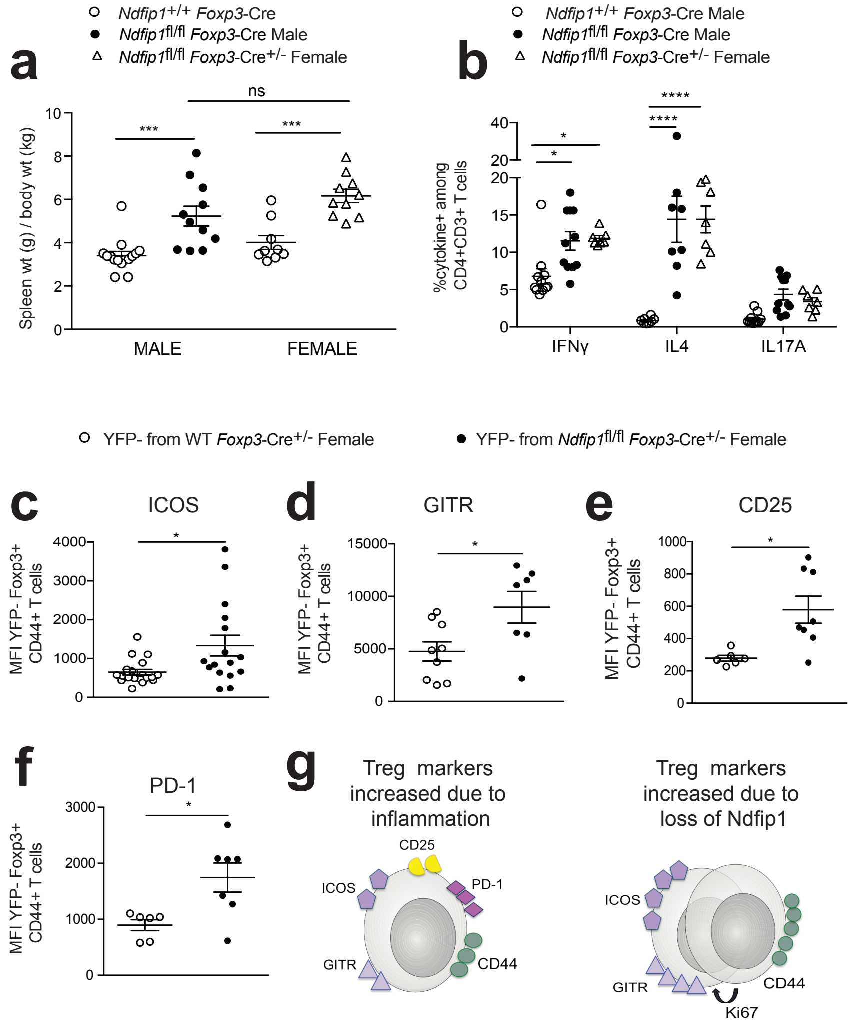

Supplementary Figure 2. *Ndfip1*$^{fl/fl}$ *Foxp3*-Cre$^{+/-}$ female animals develop disease. (**a**) Inflammation index calculated by spleen weight/body weight for male *Ndfip1*$^{fl/fl}$*Foxp3*-Cre^{+} animals, female *Ndfip1*$^{fl/fl}$ *Foxp3*-Cre$^{+/-}$ animals, compared to male *Ndfip1*$^{+/+}$*Foxp3*-Cre^{+} WT controls and female *Ndfip1*$^{+/+}$*Foxp3*-Cre$^{+/-}$ WT controls all between 8-16 weeks old. Male data from Figure 1 is added here for comparison. (**b**) *Ex vivo* PMA/ionomycin/golgi plug restimulated lung homogenates were analyzed by flow cytometry for IFNγ, IL-4 and IL-17A cytokine production from WT *Ndfip1*$^{+/+}$*Foxp3*-Cre^{+} male, *Ndfip1*$^{fl/fl}$*Foxp3*-Cre male, and *Ndfip1*$^{fl/fl}$*Foxp3*-Cre$^{+/-}$ female animals. (**c-f**) Analysis of ICOS, GITR, CD25 and PD-1 MFI are shown for the *Ndfip1*-sufficient (YFP^{-}) T_{reg} cells in unstimulated lung homogenates from *Ndfip1*$^{+/+}$*Foxp3*-Cre^{+} WT female or *Ndfip1*$^{fl/fl}$*Foxp3*-Cre$^{+/-}$ female animals. (**g**) Summary of T_{reg} cell proteins whose expression is increased by inflammation versus those which are increased directly due to loss of Ndfip1. Error bars indicate mean ± SEM. Each dot represents an individual mouse. All experiments were performed on at least two independent occasions using at least 3 female animals of each genotype. *P* value calculated by one way ANOVA (**a,b**) or unpaired *t* test (**c-f**). * $p<0.05$ ** $p<0.01$ *** $p<0.001$ ****$p<0.0001$.

Supplementary Figure 3

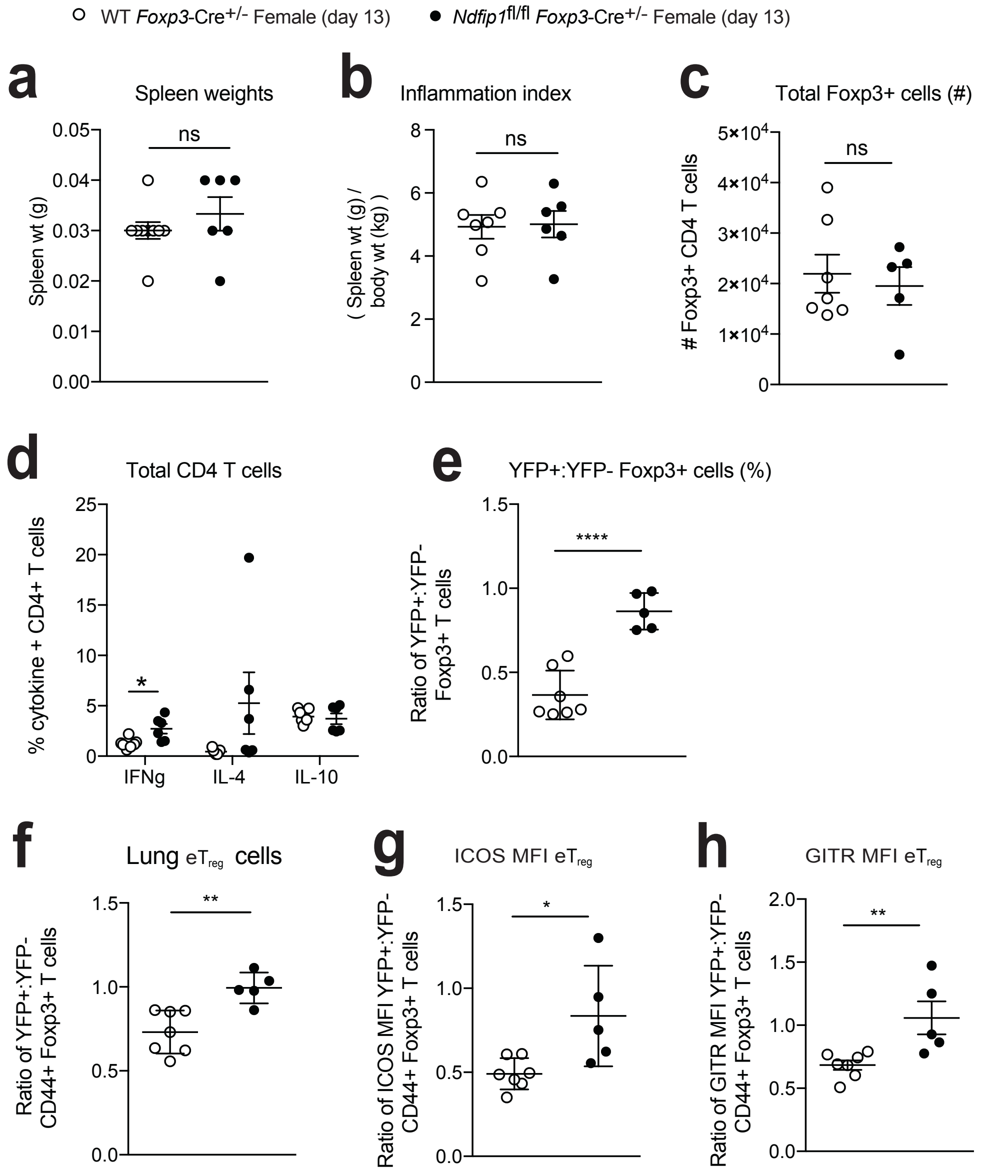

Supplementary Figure 3. ***Ndfip1*-deficient T_{reg} cells have altered phenotypes prior to inflammation onset.** (**a**) Spleen weights and (**b**) Inflammation index calculated by spleen weight/body weight for 13 day old female *Ndfip1*$^{fl/fl}$ *Foxp3*-Cre$^{+/-}$ neonates compared to age-matched *Ndfip1*$^{+/+}$*Foxp3*-Cre^{+} WT controls. (**c-d**) Lung homogenates were analysed by flow cytometry for total Foxp3^{+} cells (**c**) or stimulated *ex vivo* with PMA/ionomycin/golgi plug and analysed for total cytokine-producing CD4^{+} T cells (**d**). (**e-f**) Percentages of (**e**) Foxp3^{+} cells or (**f**) CD44^{+} eT_{reg} cells are shown as a ratio of YFP^{+}: YFP^{-} cells to normalize for effects of Cre expression (**g-h**) Analysis of ICOS (**g**) and GITR (**h**) MFI on unstimulated lung homogenates. Error bars indicate mean ± SEM. Each dot represents an individual mouse. All experiments were performed on at least two independent occasions. P value calculated by unpaired *t* test (**a-c, e-h**) or one-way ANOVA (**d**). * p<0.05 ** p<0.01 *** p<0.001 ****p<0.0001

Supplementary Figure 4

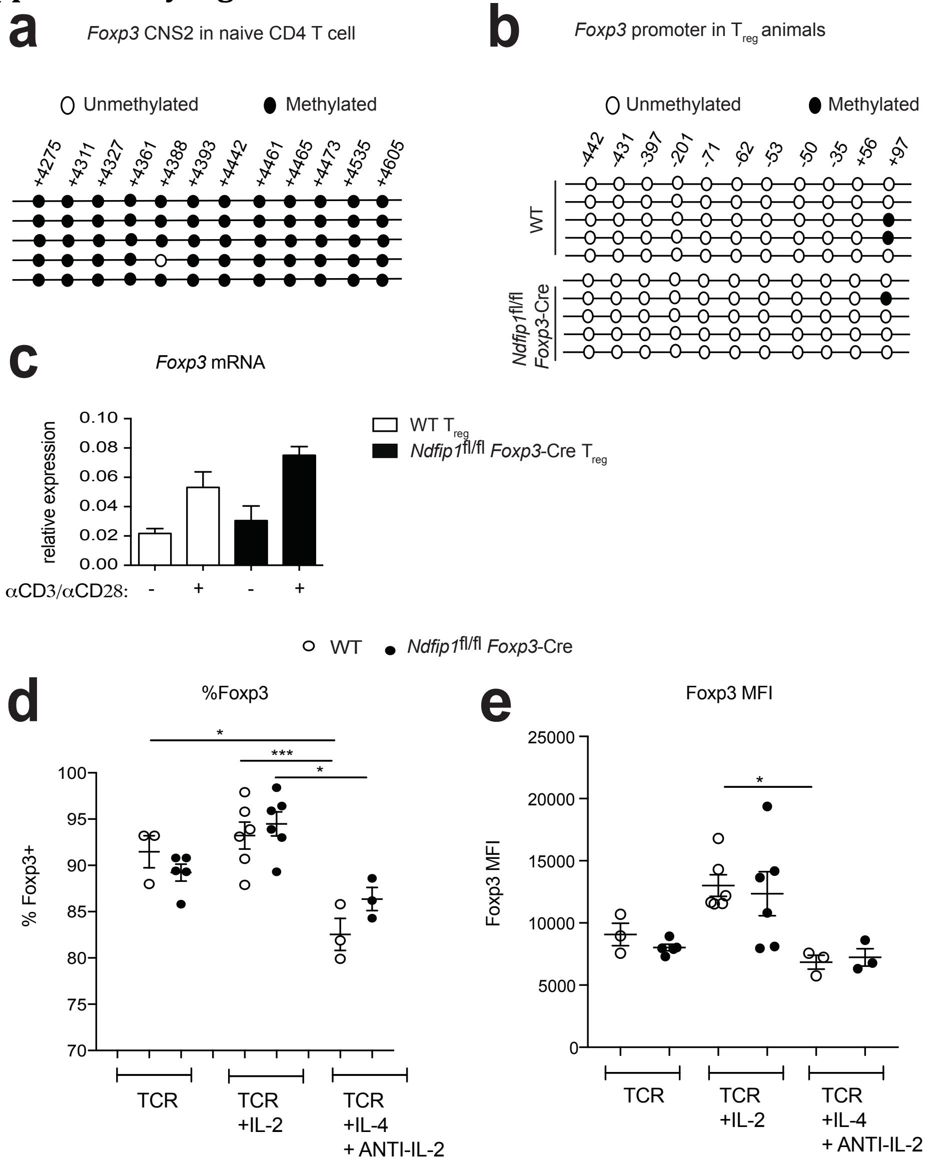

Supplementary Figure 4. *Ndfip1*-deficient T_{reg} cells show normal *Foxp3* mRNA levels but lose Foxp3 protein *in vivo*. (**a**) Analysis of the methylation status of the CNS2 region in *Foxp3*$^{-}$ (Cre negative) cells isolated from WT male animals. (**b**) Representative alleles showing methylation status of the *Foxp3* promoter region from eT_{reg} cells isolated from 9-12 week old male WT or *Ndfip1*$^{fl/fl}$*Foxp3*-Cre mice. (**c**) Expression of *Foxp3*, as measured by qPCR analysis in unstimulated or αCD3/CD28-bead-stimulated T_{reg} cells. *Foxp3* mRNA expressed as $2^{\Delta CT}$, relative to *Actb*. (**d-e**) *In vitro* percentages (**d**) and MFI (**e**) of sorted YFP^{+} Foxp3 cells are increased in response to IL-2 and decreased in response to IL-4 and anti-IL-2. *P* value calculated by one way ANOVA * p<0.05 ** p<0.01 *** p<0.001 ****p<0.0001.

Supplementary Figure 5.

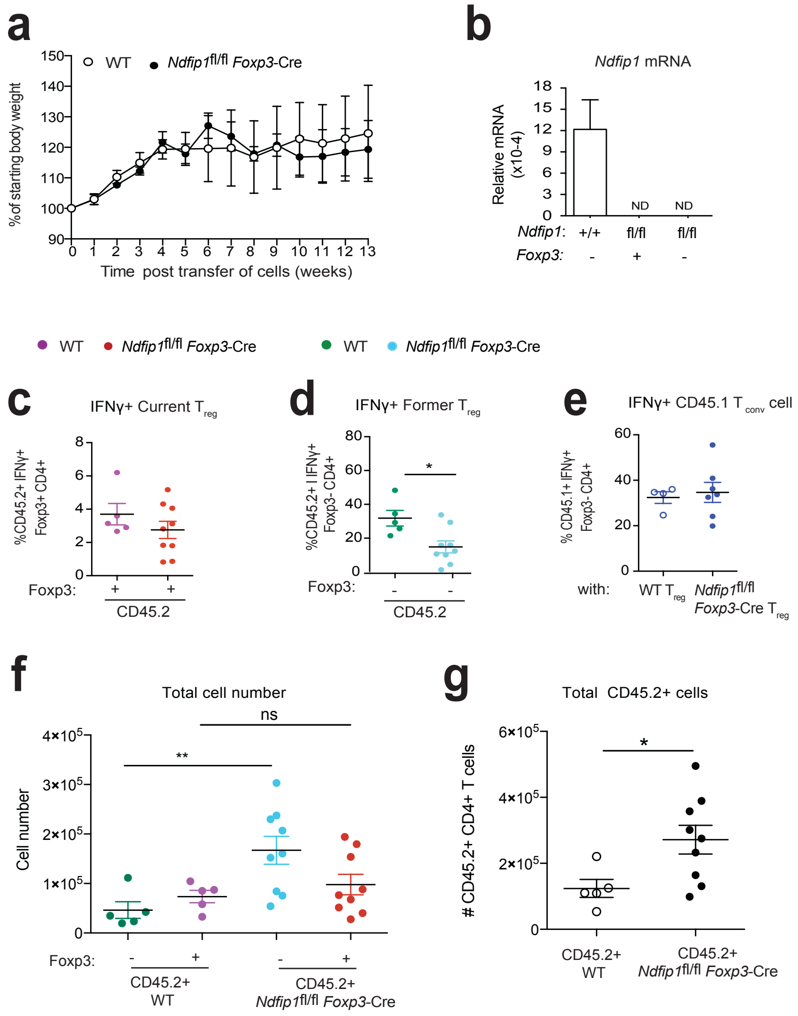

Supplementary Figure 5. *Ndfip1*-deficient T_{reg} cells are more likely than controls to lose Foxp3 *in vivo*. (**a-g**) CD45.1 $CD4^{+}$ T_{conv} cells and CD45.2 WT or *Ndfip1*$^{fl/fl}$*Foxp3*-Cre YFP^{+} T_{reg} cells were co-injected via IP at a 5 T_{conv}:1 T_{reg} ratio into *Rag1*$^{-/-}$ animals, as described in Figure 5. (**a**) Graph shows percentage change in weight over the course of the experiment. (**b**) Lung homogenates were sorted for T_{reg} cells that remained Foxp3^{+} (current T_{reg} cells), those that had become Foxp3- following transfer (former T_{reg} cells), and for CD45.1 T_{conv} cells, restimulated, and analyzed for *Ndfip1* mRNA by qPCR. (**c-e**) IFNγ production by current (**c**) and former (**d**) T_{reg} cells and from the co-transferred T_{conv} cells (**e**). (**f-g**) Absolute numbers of CD45.2^{+} current and former T_{reg} cells (**f**) or of total CD45.2^{+} cells (**g**) in recipients of WT or *Ndfip1*$^{fl/fl}$*Foxp3*-Cre T_{reg} cells. All mice were analyzed 13 weeks after the co-transfer. Error bars indicate mean ± SEM. Each dot represents an individual recipient mouse. All experiments were performed on at least two independent occasions using at least 2 animals of each genotype. *P* value was calculated by unpaired *t* tests (b-e,g) or by one-way ANOVA (f). *$p<0.05$ **$p<0.01$ ***$p<0.001$ ****$p<0.0001$

Supplementary Figure 6.

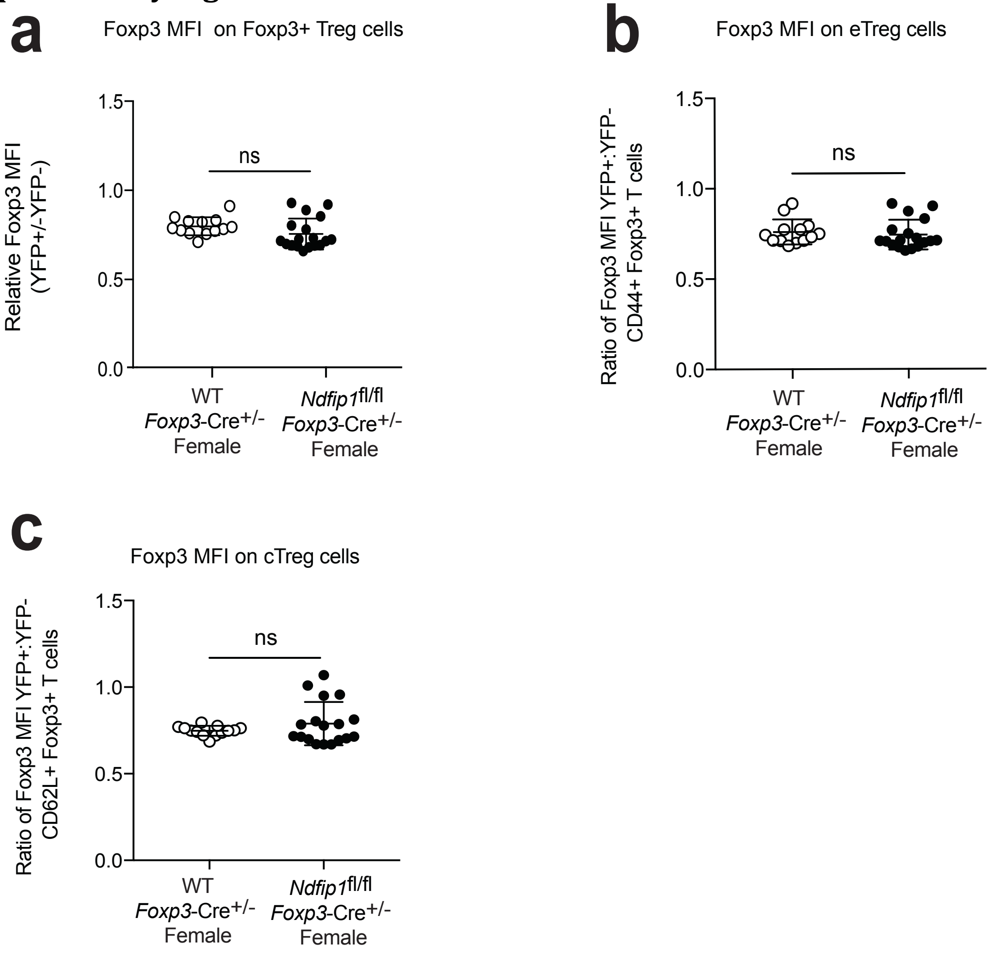

Supplementary Figure 6. *Ndfip1*$^{fl/fl}$*Foxp3*-Cre$^{+/-}$ T$_{reg}$ cells do not have decreased Foxp3 MFI.. (**a-c**) Lung homogenates, from 8-16 week old hemizygous female *Ndfip1*$^{fl/fl}$*Foxp3*-Cre$^{+/-}$ mice and *Ndfip1*$^{+/+}$*Foxp3-Cre*$^{+/-}$ controls, were analyzed *ex vivo* by flow cytometry as described in Figure 3. Data is shown for the ratio of Foxp3 MFI on: YFP^{+} to YFP^{-} Foxp3^{+} cells (**a**) YFP^{+} to YFP^{-} eT$_{reg}$ cells (**b**), or on YFP^{+} to YFP^{-} cT$_{reg}$ cells (**c**) in *Ndfip1*$^{+/+}$*Foxp3*-Cre$^{+/-}$ controls versus *Ndfip1*$^{fl/fl}$*Foxp3*-Cre$^{+/-}$ mice. *P* values determined by student's *t* test. $*p<0.05$ $**p<0.01$ $***p<0.001$ $****p<0.0001$. Each dot shows data acquired from a single female mouse. Graphs show mean ± SEM. All experiments were performed on at least two independent occasions.

Supplementary Figure 7.

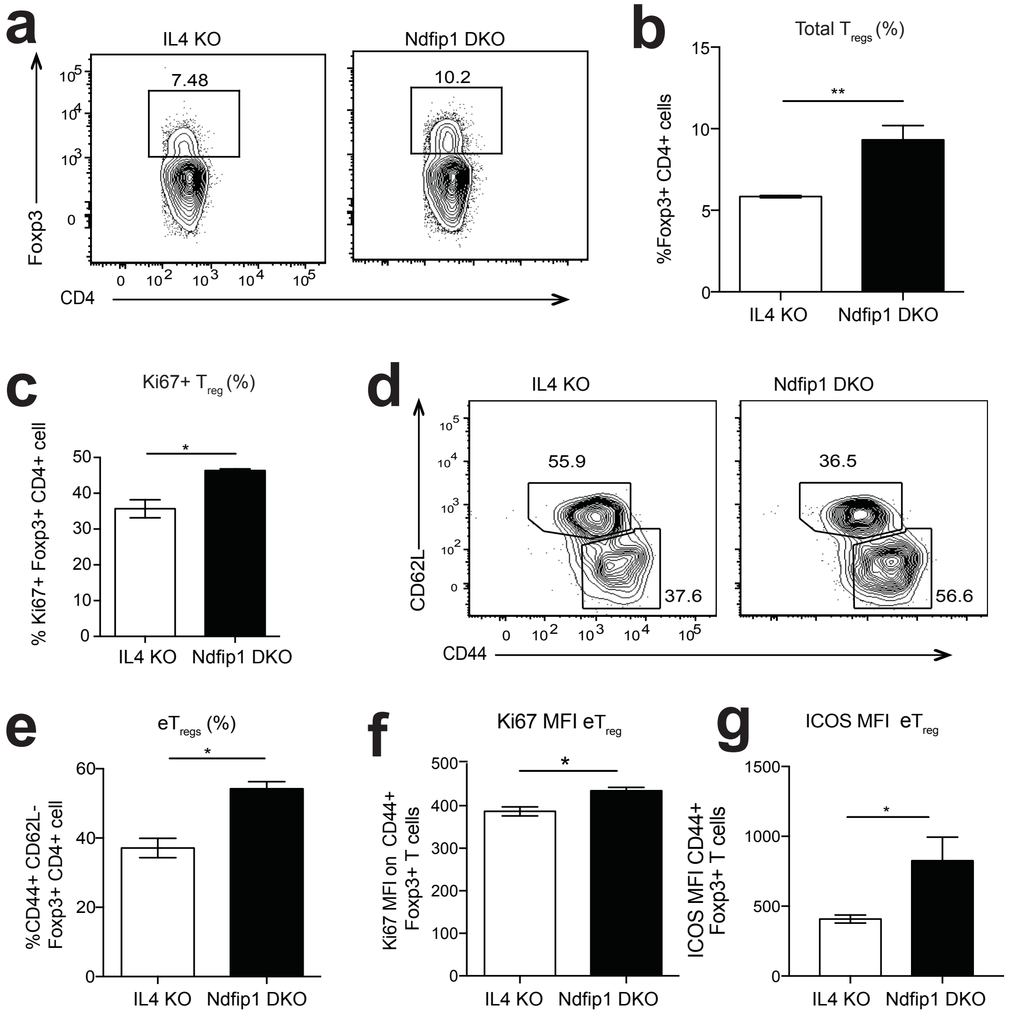

Supplementary Figure 7. IL-4 does not drive the *in vivo* fitness of *Ndfip1*-deficient T_{reg} cells. (**a-g**) Regulatory T cells from the lung of *IL-4* KO and *Ndfip1 IL-4* DKO mice were analyzed by flow cytometry *ex vivo*. (**a**) Representative flow plots of $Foxp3^+$ T_{reg} cells from lung. (**b-c**) Percentage of (**b**) total lung T_{reg} cells or (**c**) total lung Ki-67^+ T_{reg} cells. (**d**) Representative flow plots of eT_{reg} and cT_{reg} cells, identified by CD44 and CD62L staining of lung homogenate. Previously gated on Foxp3. (**e**) Summary of percentages of eT_{reg} cells from (d). (**f-g)** $CD44^+$ lung $Foxp3^+$ eT_{reg} cells were analyzed for expression of the proliferation marker, Ki-67 (**f**) or ICOS (**g**). Error bars indicate mean ± SEM. N=2-4 (age-matched male or female) animals per genotype in one experiment. P values determined by student's *t* test * $p<0.05$ ** $p<0.01$.

Supplementary Figure 8.

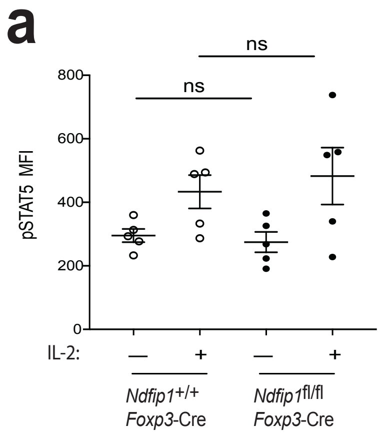

Supplementary Figure 8. *Ndfip1*-deficient T_{reg} cells do not show increased IL-2-induced STAT5 phosphorylation. Sorted YFP^+ total T_{reg} cells were rested overnight without TCR or cytokines. The MFI for STAT5 phosphorylation as measured by flow cytometry, is shown for the T_{reg} cells with or without addition of 119 U/ml (~50ng/ml) of rhIL-2 for 30minutes in a 37°C incubator. Each dot represents data acquired from a single mouse (male or female, between 9-16 weeks old). Graphs show mean ± SEM and represent data from at least two independent occasions.

Supplementary Figure 9

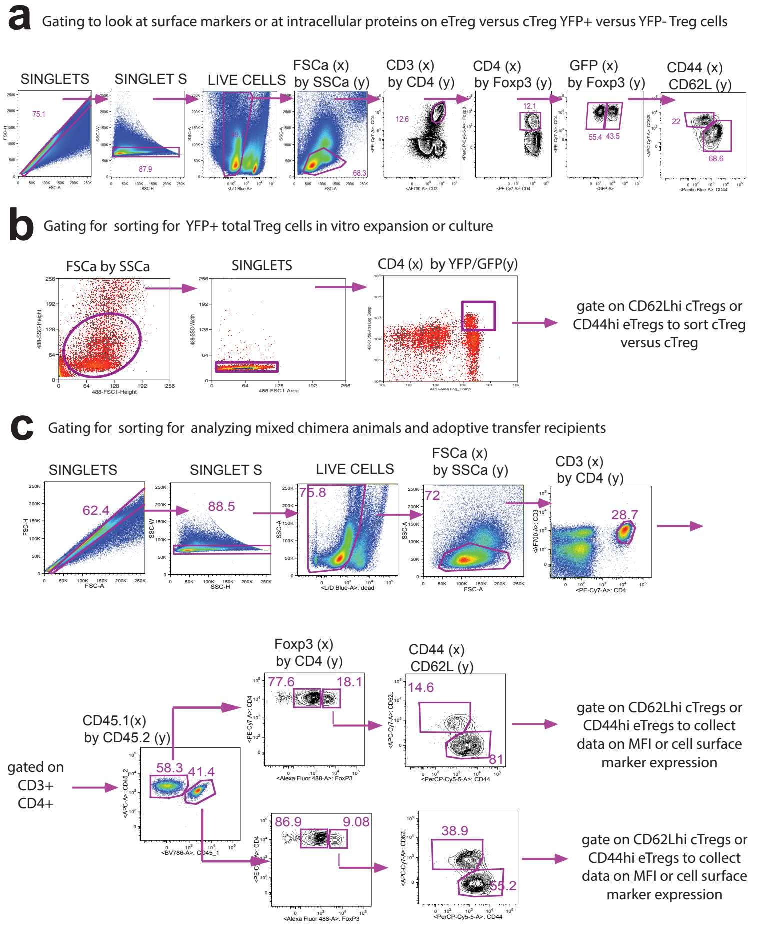

Supplementary Figure 9. Flow cytometry gating schemes. (**a**) Gating scheme for analyzing T_{reg} cell surface proteins and intracellular proteins in female *Ndfip1*$^{+/+}$*Foxp3*-Cre$^{+/-}$ controls and *Ndfip1*$^{fl/fl}$*Foxp3*-Cre$^{+/-}$ mice shown in Figures 3, Supplementary Figure 2, and Supplementary Figure 3. Male animals were gated similarly, omitting the GFP by Foxp3 step, since all Foxp3^{+} T_{reg} cells in male animals are YFP^{+}. YFP was detected using an anti YFP/GFP antibody. The gating schema for males was used for Figure 1e-f, Figure 2, Figure 4,Figure 5, and Figure 8. (**b**) Gating scheme for sorting total CD4^{+} YFP^{+} Treg cells. Total T_{reg} cells were sorted for Figure 1a, Figure 4c, Figure 7, Figure 8, Supplementary Figure 4c, and Supplementary Figure 5b. To sort CD62L^{lo} CD44^{+} eT_{reg} and CD62L^{hi} CD44^{-} cT_{reg} cells, CD4^{+} YFP^{+} cells were further separated by CD44 and CD62L staining as shown in (a). cT_{reg} versus eT_{reg} YFP^{+} Foxp3^{+} cells were sorted for Figure 5a-b, Figure 6, Figure 7, and Figure 8. (**c**) Gating schema for mixed chimera experiments and adoptive transfer experiments. In the mixed chimera experiments in Figure 4, congenically marked *Ndfip1*$^{+/+}$*Foxp3*-Cre WT cells (CD45.1^{+} or CD45.1^{+}/CD45.2^{+}) were compared to CD45.2^{+} *Ndfip1*$^{fl/fl}$*Foxp3*-Cre cells. In the adoptive transfer experiments for Figure 5 and Supplementary Figure 5, WT T_{conv} cells (CD45.1^{+}/CD45.2^{+}) were co-transferred and compared to either CD45.2^{+} *Ndfip1*$^{+/+}$*Foxp3*-Cre WT Treg cells or CD45.2^{+} *Ndfip1*$^{fl/fl}$*Foxp3*-Cre Treg cells.
